# An examination of the internal consistency and structure of the Statistical Anxiety Rating Scale (STARS)

**DOI:** 10.1371/journal.pone.0194195

**Published:** 2018-03-14

**Authors:** Peter K. H. Chew, Denise B. Dillon, Anne L. Swinbourne

**Affiliations:** 1 College of Healthcare Sciences, James Cook University, Singapore, Singapore; 2 College of Healthcare Sciences, James Cook University, Townsville, Queensland, Australia; California State University Dominguez Hills, College of Health, Human Services and Nursing, Social Work Department - MSW Program, UNITED STATES

## Abstract

The purpose of the current study was to examine the internal consistency and structure of the English version of the Statistical Anxiety Rating Scale (STARS). Participants were 202 (79% females) psychology undergraduates was recruited from James Cook University’s Singapore (71%) and Australia (29%) campuses. Acceptable internal consistency reliabilities, ranging from .81 to .94, were found in this sample. Approximate fit indices suggest that a correlated six first-order factor model best describes the data in contrast to theoretical considerations suggesting that a six factor model with two correlated superordinate factors (i.e., statistics anxiety and attitudes toward statistics) best describes the data. Researchers are recommended to use part one of the STARS to assess statistics anxiety and part two to assess attitudes toward statistics.

## Introduction

Cruise, Cash, and Bolton [1] defined statistics anxiety “as the feelings of anxiety encountered when taking a statistics course or doing statistical analyses” (p. 92). Cruise et al. developed the Statistical Anxiety Rating Scale, commonly known as the STARS, to measure statistics anxiety. An initial 89-item pilot instrument was completed by 1150 participants in the USA and the data subjected to factor analysis using the Principal Components Method with varimax rotation. Results indicated that the rotation of 51 items on six factors yielded the most interpretable structure. The six factors were: (a) Interpretation Anxiety, (b) Test and Class Anxiety, (c) Fear of Asking for Help, (d) Worth of Statistics, (e) Computation Self-Concept, and (f) Fear of Statistics Teachers.

‘Interpretation Anxiety’ refers to the feelings of anxiety encountered when interpreting statistical data. The ‘Test and Class Anxiety’ factor encompasses the anxiety involved when attending a statistics class or when taking a statistics test. ‘Fear of Asking for Help’ assesses the anxiety experienced when seeking help. ‘Worth of Statistics’ relates to an individual’s perception of the relevance of statistics to the individual. ‘Computation Self-Concept’ relates to an individual’s self-perception of his or her ability to understand and calculate statistics. Lastly, ‘Fear of Statistics Teachers’ refers to an individual’s perception of the statistics teacher.

The 51-item STARS consists of two parts [1]. Part one consists of 23 items which assess statistics anxiety associated with situations where students have contact with statistics and it includes the following factors: (a) Interpretation Anxiety, (b) Test and Class Anxiety, and (c) Fear of Asking for Help. Individuals respond on a 5-point Likert scale that ranges from 1 = *No Anxiety* to 5 = *Strong Anxiety*. Part two consists of 28 items that measure the level of agreement with various statements about statistics and statistics teachers and it includes the following factors: (d) Worth of Statistics, (e) Computation Self-Concept, and (f) Fear of Statistics Teachers. Responses are made on a 5-point Likert scale that ranges from 1 = *Strongly Disagree* to 5 = *Strongly Agree*.

Despite the existence of newer measures of statistics anxiety, such as the Statistical Anxiety Scale [2], the STARS [1] has been used extensively by researchers due to the superiority of its reliability and validity data as compared with other measures [3]. The psychometric properties of the STARS have been examined and empirical support has been found for the six-factor structure in several studies using student populations in the South Africa [4], the UK [5], China [6], Austria [7], and the USA [8]. The internal consistencies of the STARS reported by these studies are summarized in Table 1. Furthermore, Keeley, Zayac, and Correia [9] reported two-week test-retest reliabilities that ranged from .76 to .87 and four-months test-retest reliabilities that ranged from .41 to .74 (*n* = 83). More recently, Papousek et al. [7] reported five-month test-retest reliabilities that ranged from .49 to .78 (*n* = 89). With regard to validity, despite providing support for the six-factor structure, several researchers have argued that the STARS assesses both statistics anxiety and attitudes toward statistics rather than only statistics anxiety [5,7,8].

**Table 1:** Internal consistency reliability coefficients (Cronbach’s alpha) of the STARS among six studies.

Factors	USA [1]	South Africa [4]	UK[5]	China [6]	Austria [7]	USA [8]
	(*n* = 1150)	(*n* = 169)	(*n* = 650)	(*n* = 201)	(*n* = 400)	(*n* = 517)
Interpretation	.89	.77	.87	.86	.88	.92
Test	.91	.77	.87	.85	.87	.90
Fear	.85	.68	.83	.72	.86	.88
Worth	.94	.86	.94	.91	.94	.95
Self-Concept	.88	.81	.87	.74	.86	.89
Teachers	.80	.74	.83	.69	.80	.82
Total Scale	-	.92	-	.94	.96	-

Interpretation = Interpretation Anxiety; Test = Test and Class Anxiety; Fear = Fear of Asking for Help; Worth = Worth of Statistics; Self-Concept = Computation Self-Concept; Teachers = Fear of Statistics Teachers.

Hanna et al. [5] examined the structure of the STARS with a sample of 650 undergraduate psychology students in the UK and reported that a correlated six first-order factor model explained the data better than a six factor model with one superordinate factor. The results were unexpected because the latter model should be a better model if all six factors of the STARS assess statistics anxiety alone. For example, all six factors should load on a single superordinate factor (i.e., statistics anxiety) if the STARS assesses statistics anxiety only. Instead, the results suggest that while the six factors are correlated, they might assess a construct more multifaceted than statistics anxiety. Furthermore, Hanna et al. noted that many items and factors of the STARS appear to assess related concepts of statistics anxiety, such as attitudes toward statistics. Based on these findings, Hanna et al. suggested replacing the term “statistics anxiety” with a more appropriate label such as “statistical attitudes and anxiety”.

Subsequently, Papousek et al. [7] translated the STARS to German and examined its structure with a sample of 400 undergraduate students in Austria. Papousek et al. argued that the two-part nature of the STARS, as well as the different labels assigned to the Likert scales (anxiety vs. agreement), suggests that part one of the STARS assesses statistics anxiety and part two assesses attitudes toward statistics. Papousek et al. extended the work of Hanna et al. [5] by including a six-factor model with two superordinate factors representing three factors each: statistics anxiety (Interpretation Anxiety, Test and Class Anxiety, and Fear of Asking for Help) and attitudes toward statistics (Worth of Statistics, Computation Self-Concept, and Fear of Statistics Teachers). Due to the use of multiple fit indices, two models were found to be equally acceptable: a modified correlated six first-order factor model (13 error correlations were specified and item 47 was reassigned to load on another factor) and the modified six factor model with two superordinate factors. However, Papousek et al. provided support for the latter model by demonstrating differential validity between statistics anxiety and attitudes toward statistics in subsequent validation studies. Nevertheless, it should be noted that the conclusions were based on the German adaptation of the STARS.

Finally, DeVaney [8] examined the structure of the STARS with a sample of 517 graduate students enrolled in online introductory statistics course in the USA. Three models were specified: An uncorrelated six first-order factor model (Model 1), a correlated six first-order factor model (Model 2), and a six factor model with two correlated superordinate factors (i.e., statistics anxiety and attitudes toward statistics; Model 3). The results provided support for the use of both Model 2 and Model 3, with the latter model performing slightly better on the parsimony fit indices. Despite using the English version of the STARS, it should be noted that a six factor model with one superordinate factor (i.e., statistics anxiety only) was not examined in the study. This exclusion precluded a comparison of the constructs assessed by the instrument (i.e., statistics anxiety only or statistics anxiety and attitudes toward statistics).

Given the popularity of the STARS, it is important for researchers to be aware of the constructs assessed by the instrument. A clarification of the structure of the STARS and a distinction between statistics anxiety and attitudes toward statistics offers researchers two major advantages. First, researchers can gain more insights into their data. For example, Bell [10] reported that non-traditional university students (defined as students aged 25 years and above) scored higher on the Test and Class Anxiety factor whereas traditional students (below the age of 25 years) scored higher on the Worth of Statistics factor of the STARS. As high scores indicate higher anxiety, the results suggest that both groups of students experience statistics anxiety, but that anxiety appears to be associated with different factors. However, if the STARS assesses both anxiety and attitudes (with high scores on Worth of Statistics indicating more negative attitudes), this finding could be reinterpreted to mean that non-traditional students had higher statistics anxiety but understood the importance of statistics than did traditional students. Second, researchers can prevent multicollinearity when both variables are studied concurrently. For example, Nasser [11] removed statistics anxiety from a model of statistics achievement due to multicollinearity with attitudes toward statistics. This precluded an investigation on the relative importance of each variable in predicting statistics achievement.

Although a few studies have utilized confirmatory factor analysis to examine the construct validity of the STARS, there are limitations associated with these studies [5,7,8]. Both Hanna et al. [5] and DeVaney [8] used the English version of the STARS. However, Hanna et al. did not include a six factor model with two correlated superordinate factors whereas DeVaney did not include a six factor model with one superordinate factor. In contrast, although Papousek et al. [7] examined both models concurrently, the study used a German adaptation of the STARS and the conclusions might not generalize to the English version of the STARS. Accordingly, the purpose of the current study is to bridge the research gap by examining the internal consistency and structure of the English version of the STARS. Three models are specified and evaluated: a correlated six first-order factor model (Model 1), a six factor model with one superordinate factor (i.e., statistics anxiety only) (Model 2), and a six factor model with two correlated superordinate factors (Model 3). For Model 3, it is hypothesized that part one of the STARS (Interpretation Anxiety, Test and Class Anxiety, and Fear of Asking for Help factors) will load on one superordinate factor (i.e., statistics anxiety) and part two of the STARS (Worth of statistics, Computation Self-Concept, and Fear of Statistics Teachers factors) will load on another superordinate factor (i.e., negative attitudes toward statistics). It is hypothesized that Model 3 will best represent the data from the current sample.

## Method

### Participants

A convenience sample of 202 (79% females) psychology undergraduates was recruited from James Cook University’s Singapore (71%) and Australia (29%) campuses. Their ages ranged from 17 to 54 years (*M* = 23.72, *SD* = 7.18). The predominantly female sample was consistent with the gender distribution of the psychology undergraduate population in Singapore and Australia. Participants were either currently enrolled in a statistics course (74%) or had completed at least one statistics course but were not currently enrolled in a statistics course (26%). Barrett [12] recommends a minimum sample size of 200 for Confirmatory Factor Analysis/Structural Equation Modelling.

### Instruments

#### STARS

The basic structure and response format of the STARS have been described earlier. Appropriate item scores are summed for each factor, with higher scores indicating higher levels of statistics anxiety.

#### Statistical anxiety scale (SAS)

The SAS is a 24-item instrument designed to assess three factors of statistics anxiety: (a) Examination Anxiety, (b) Asking for Help Anxiety, and (c) Interpretation Anxiety [2]. Individuals respond on a 5-point Likert scale that ranges from 1 = *No Anxiety* to 5 = *Considerable Anxiety*. Appropriate item scores are summed for each factor, with higher scores indicating higher levels of statistics anxiety. Vigil-Colet et al. [2] reported internal consistency ranging from.82 to .92 for the subscales and .91 for the total scale (*n* = 159). The three-factor structure has been confirmed in at least one psychometric study [13].

#### Attitudes toward statistics scale (ATS)

The ATS is a 29-item instrument designed to assess two aspects of an individual’s attitudes toward statistics: (a) Attitudes toward Field and (b) Attitudes toward Course [14]. Responses are made on a 5-point Likert scale that ranges from 1 = *Strongly Disagree* to 5 = *Strongly Agree*. Fourteen negatively worded items are reverse scored and the appropriate item scores are summed for each factor and for the total scale. Higher scores indicate higher levels of positive attitudes toward statistics. Wise [14] reported internal consistency of .92 and .90, and two week test-retest reliability of .82 and .91, for the Attitudes toward Field subscale and the Attitudes toward Course subscale, respectively (*n* = 92). The two-factor structure has been confirmed in other factor-analytic studies [15,16].

### Procedure

A link to the online study was made available to potential participants. Participants were presented with an information page which describes the study and the type of information being requested from them. Subsequently, participants provided informed consent by clicking ‘Next’ to proceed to the study. Participants completed a demographics form, the STARS [1], the SAS [2], and the ATS [14]. Each instrument took about 10 minutes to complete. Both the STARS and the SAS are measures of statistics anxiety whereas the ATS is a measure of attitudes toward statistics. All instruments were administered online and counterbalanced to control for order effects. Participants either received extra course credit or were entered into a lucky draw for a chance to win an iPod shuffle. This procedure was approved by James Cook University’s Human Research Ethics Committee (Approval number H4761).

### Data analysis

There are three scenarios in the general strategic framework for testing structural equation models: (a) strictly confirmatory, (b) alternative models, and (c) model generating [17]. The model generating scenario is currently the most common approach [18]. In this scenario, an initial model is specified and evaluated against a set of fit indices. If the model represents a poor fit to the data, the researcher identifies the source of misfit and modifies the model. For example, Papousek et al. [7] specified 13 error correlations and reassigned an item to another factor to improve model fit. Nevertheless, criticisms have been directed at some aspects of the model generating scenario.

Approximate fit indices were originally developed to indicate degree of model fit to data. However, recommended cutoff values of these indices have been elevated to golden rules, resulting in a binary decision (fit/no fit) of model fit [19]. For example, the recommended cutoff value of the Comparative Fit Index (CFI) is .95 [20], and models with a value of more than .95 are considered a good fit. Consequently, Barrett [12] recommends banning the use of such fit indices. Another criticism deals with the non-generalizability of modifications [21]. Because modifications (e.g., error correlations) are data driven, the modifications might not generalize to samples in other studies or to the population. Therefore, the current study uses the alternative models scenario to test structural equation models [17,21,22].

In the alternative models scenario, several competing models, grounded in theory, are specified and evaluated [18]. Based on fit indices, one model would be selected as the best model to represent the data. The Linhart and Zucchini’s [23] bootstrap approach to model comparison is used in this study. The bootstrap approach is summarized in four steps: (a) generate multiple bootstrap samples from the current sample, (b) fit every model to every bootstrap sample and calculate the discrepancy of the implied moments between the sample and population, (c) calculate the average discrepancy across bootstrap samples for each model, and (d) select the model with the lowest average discrepancy [24].

Additionally, several fit indices were used to aid interpretation. These indices are the Browne-Cudeck Criterion (BCC), the Expected Cross-Validation Index (ECVI) [25], and the Consistent Akaike’s Information Criterion (CAIC) [26]. These indices do not have recommended cutoff values; instead, they are compared across models, with lower values indicating better model fit relative to other competing models.

Lastly, the theoretical appropriateness of the models was considered [24]. Indeed, “the assessment of model adequacy should be a multifaceted enterprise comprising consideration of model fit, empirical adequacy and substantive meaningfulness” [27]. Theoretical appropriateness of the models was in this instance evaluated by examining convergent and divergent validity using the SAS [2] and the ATS [14].

## Results

All results were analyzed using SPSS and AMOS version 16.0 with the alpha level set at .05. Preliminary analyses suggest that females (*M* = 19.43, *SD* = 7.23) had higher scores on the Computation Self-Concept factor than males [*M* = 16.35, *SD* = 5.86, *t*(80.09) = -2.90, *p* < .01]. Additionally, participants from the Singapore campus (*M* = 32.73, *SD* = 8.16) had higher scores on the Interpretation Anxiety factor than their counterparts from the Australian campuses [*M* = 27.10, *SD* = 9.55, *t*(200) = -4.23, *p* < .001]. However, the sample size was not large enough to permit separate investigations. Thus, the results were collapsed across gender and campuses. Internal consistencies, means, and standard deviations of the STARS [1], the SAS [2], and the ATS [14] are presented in Table 2. Cronbach’s alphas of the STARS ranged from .81 to .94, which was well above the acceptable alpha of .70 [28]. The intercorrelations between factors of the STARS are presented in Table 3.

**Table 2:** Internal consistencies (Cronbach’s alpha), means, and standard deviations of the STARS, the SAS, and the ATS.

STARS	Cronbach’s alpha	*M*	*SD*	No. of Items	*M* / No. of Items
Interpretation Anxiety	.91	31.08	8.94	11	2.83
Test and Class Anxiety	.89	28.71	6.51	8	3.59
Fear of Asking for Help	.88	9.93	4.07	4	2.48
Worth of Statistics	.94	39.09	13.49	16	2.44
Computation Self-Concept	.90	18.77	7.06	7	2.68
Fear of Statistics Teachers	.81	11.41	4.20	5	2.28
**SAS**					
Examination Anxiety	.89	32.93	5.69	8	4.17
Asking for Help Anxiety	.95	20.49	8.21	8	2.56
Interpretation Anxiety	.89	21.18	6.71	8	2.65
**ATS**					
Attitudes toward Field	.91	72.67	11.58	20	3.63
Attitudes toward Course	.91	26.78	8.35	9	2.98

**Table 3:** Intercorrelations between factors of the STARS.

	1	2	3	4	5	6
1. Interpretation Anxiety	-					
2. Test and Class Anxiety	.62*	-				
3. Fear of Asking for Help	.69*	.48*	-			
4. Worth of Statistics	.44*	.35*	.43*	-		
5. Computation Self-Concept	.51*	.59*	.48*	.74*	-	
6. Fear of Statistics Teachers	.40*	.29*	.42*	.68*	.61*	-

*p < .01.

**Fig 1:**
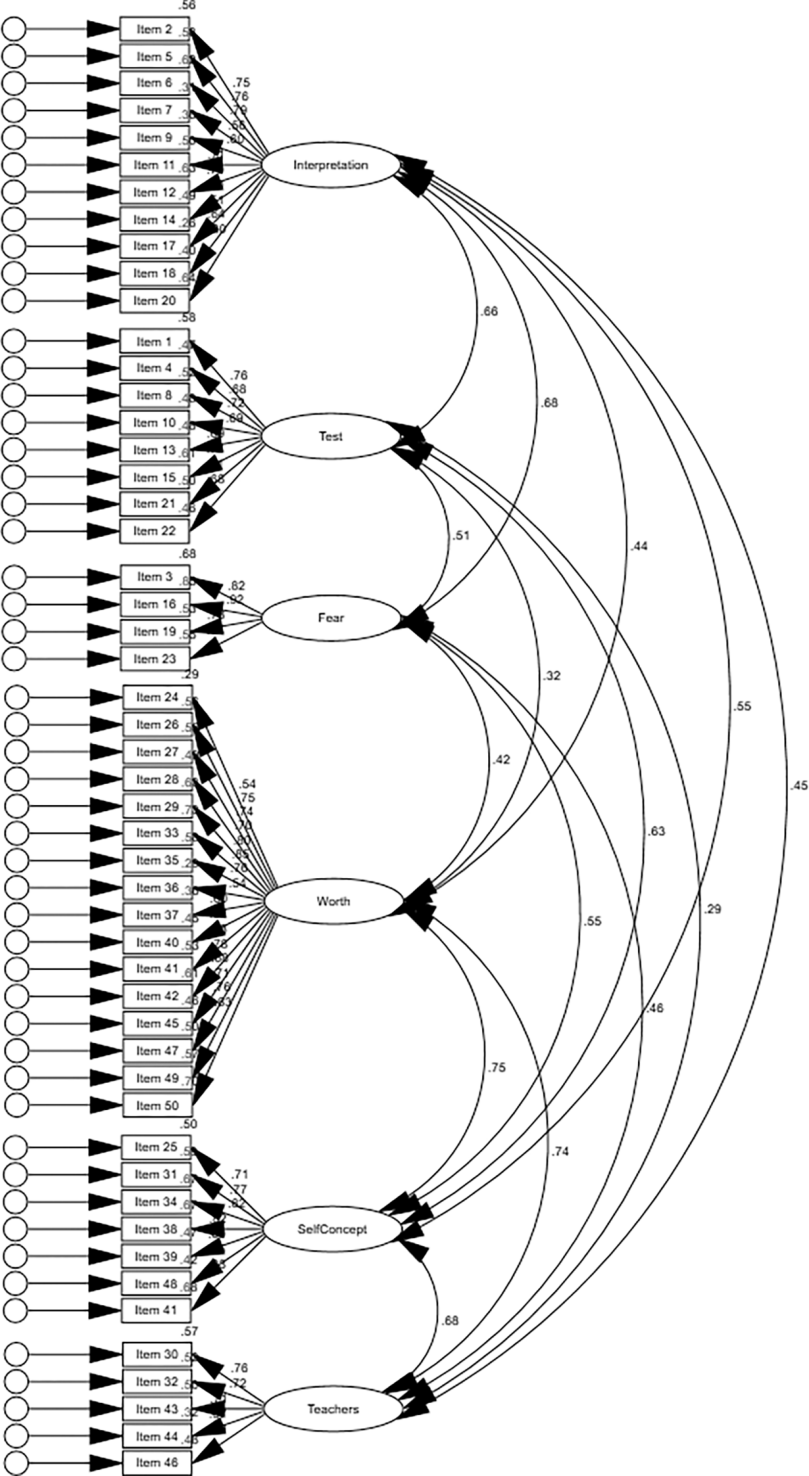
Standardized estimates for Model 1.

**Fig 2:**
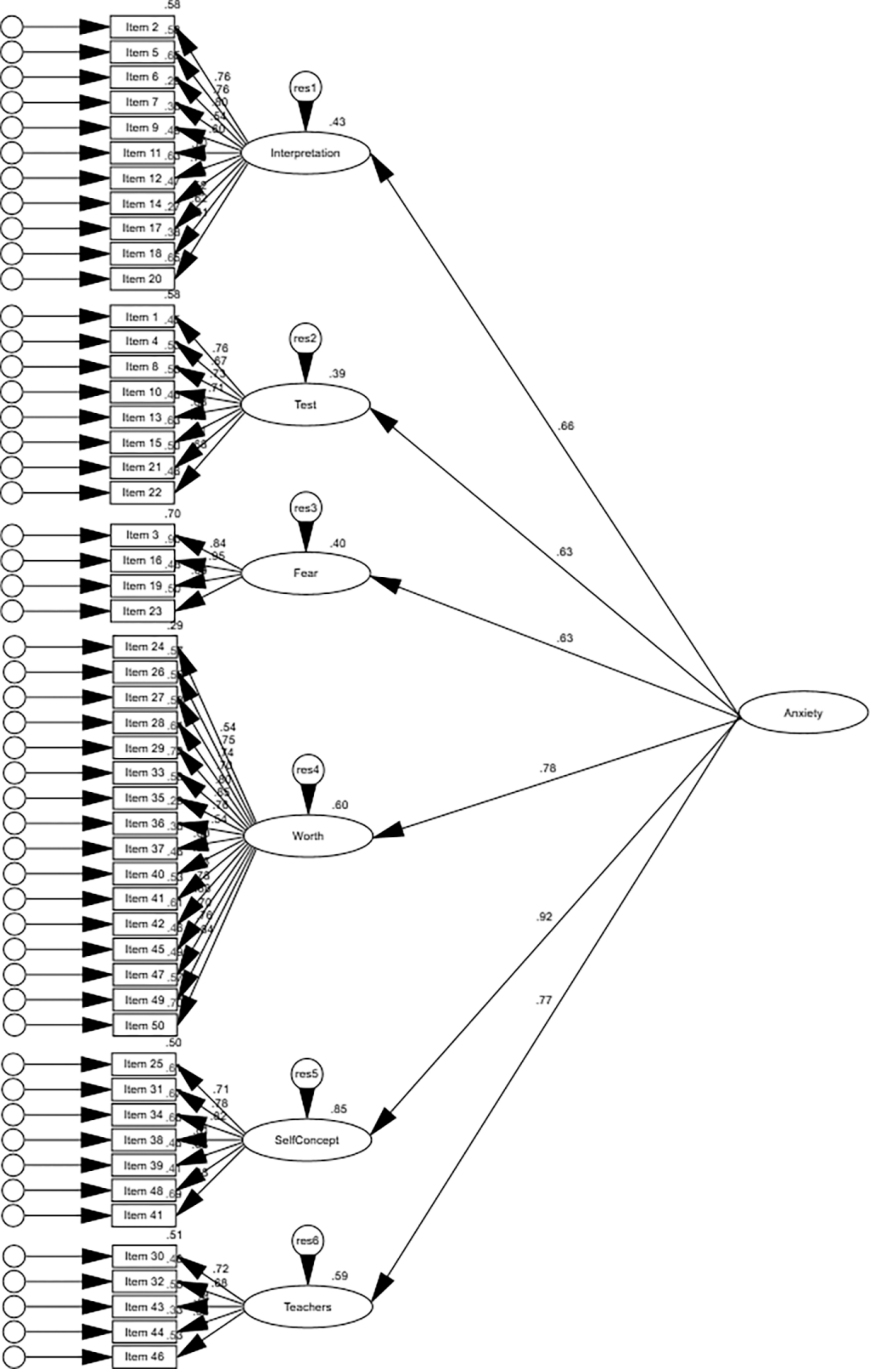
Standardized estimates for Model 2.

**Fig 3:**
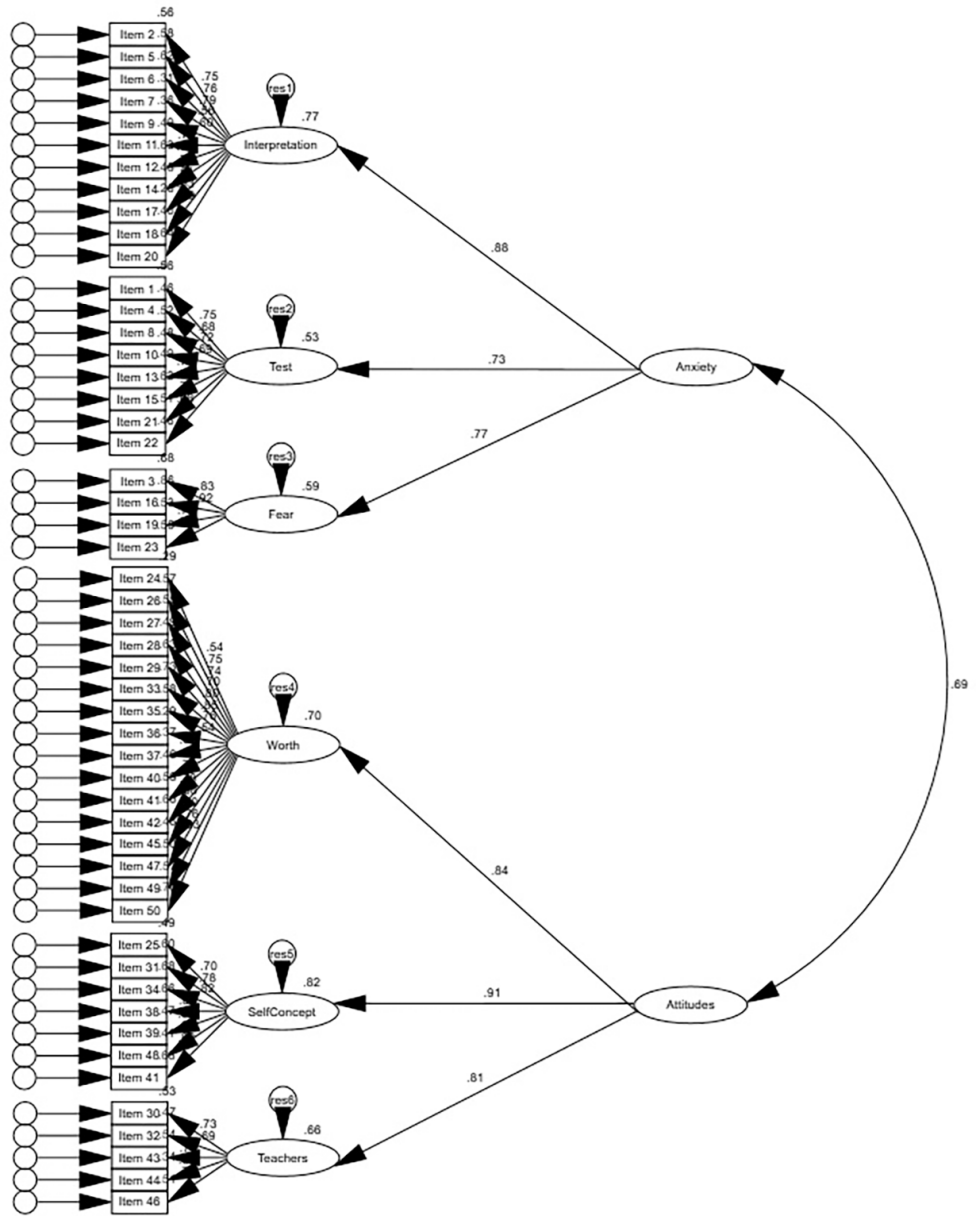
Standardized estimates for Model 3.

To evaluate the structure of the STARS [1], the following models were specified and evaluated: a correlated six first-order factor model (Model 1), a six-factor model with one superordinate factor (i.e., statistics anxiety only) (Model 2), and a six-factor model with two correlated superordinate factors (i.e., statistics anxiety and negative attitudes toward statistics) (Model 3). Bootstrapping was used with 1000 bootstrap samples and the results are presented in Table 4. Model 1 had the lowest mean discrepancy and fit indices values, followed closely by Model 3 and lastly, Model 2. Hence, the fit indices suggest Model 1 to be the best fit to the data of the three models tested (see Figs 1–3 for the standardized estimates of all three models, respectively). The nested χ^2^ difference test was conducted to compare the remaining models. Model 3 (χ^2^_(1217)_ = 2760.41) was a better fit to the data than Model 2 (χ^2^_(1218)_ = 2824.85), Δ χ^2^_(1)_ = 64.44, *p* < .001.

**Table 4:** Average mean discrepancies and fit indices for three competing models of the STARS.

Model	Failures	Mean Discrepancy	BCC	CAIC	ECVI
1	0	2907.93	3023.56	3445.96	14.64
2	0	3012.55	3116.23	3506.14	15.13
3	0	2951.16	3054.49	3448.01	14.82

Model 1 = correlated six first-order model; Model 2 = six-factor model with one superordinate factor; Model 3 = six-factor model with two correlated superordinate factors; BCC = Browne-Cudeck Criterion; CAIC = Consistent Akaike’s Information Criterion; ECVI = Expected Cross-Validation Index.

### Convergent and divergent validity

The theoretical appropriateness of the models was evaluated by examining convergent and divergent validity of the factors. The hypothesized superordinate factors of the STARS were derived by summing scores on the respective factors: (a) STARS-Anxiety (Interpretation Anxiety, Test and Class Anxiety, and Fear of Asking for Help factors), (b) STARS-Negative Attitudes (Worth of Statistics, Computation Self-Concept, and Fear of Statistics Teachers factors), and (c) STARS-Total Scale (all six factors). SAS-Anxiety was derived by summing scores from the three factors of the SAS [2] and ATS-Positive attitudes was derived by summing scores from the two factors of the ATS [14]. Table 5 presents the correlations between these variables.

**Table 5:** Correlations between the six factors of the STARS, STARS-Anxiety, STARS-Negative Attitudes, STARS-Total Scale, SAS-Anxiety, and ATS-Positive attitudes.

STARS	SAS-Anxiety^a^	ATS-Positive Attitudes^b^
**Model 1**		
**Part one**		
Interpretation Anxiety	.78*	-.37*
Test and Class Anxiety	.73*	-.36*
Fear of Asking for Help	.79*	-.37*
**Part two**		
Worth of Statistics	.48*	-.79*
Computation Self-Concept	.63*	-.69*
Fear of Statistics Teachers	.46*	-.53*
**Model 2**		
STARS-Total Scale	.80*	-.72*
**Model 3**		
STARS-Anxiety	.89*	-.42*
STARS-Negative Attitudes	.57*	-.79*

^a^SAS-Anxiety assessed using the SAS [2].

^b^ATS-Positive attitudes assessed using the ATS [14].

*p < .01.

At least one factor in each model was highly correlated with ATS-Positive Attitudes. Model 3 was the only model to discriminate between anxiety and attitudes: STARS-Anxiety had a larger correlation with SAS-Anxiety than ATS-Positive Attitudes whereas STARS-Negative Attitudes had a larger correlation with ATS-Positive Attitudes than SAS-Anxiety. Therefore, theoretical considerations suggest Model 3 best describe the data compared to competing models.

## Discussion

The purpose of the study was to examine the internal consistency and structure of the English version of the STARS. Consistent with previous studies [5,7,8], acceptable internal consistency reliabilities were found in the current study. For example, Papousek et al. [7] reported internal consistency which ranged from .80 to .96 whereas the current study reported internal consistency which ranged from .81 to .94. With regard to the structure of the STARS, it was hypothesized that Model 3 would best represent the data from the current sample. The results provided partial support for the hypothesis. Although the fit indices suggested that Model 1 provided the best fit to the data, theoretical considerations suggested that Model 3 best describe the data.

The results were consistent with previous studies that indicate that the STARS assesses a construct broader than statistics anxiety [5,7,8]. The fit indices showed that Model 2 represented a poor fit to the data compared to Model 1 and Model 3. More important, the use of only one superordinate factor (i.e., statistics anxiety) did not discriminate between anxiety and attitudes. The total scale of the STARS had large correlations with statistics anxiety and attitudes toward statistics. This would result in multicollinearity in studies where both variables are examined concurrently [11]. Thus, researchers should not use the STARS as a measure of statistics anxiety.

The results were also consistent with previous studies that found both the correlated six first-order factor model and the six-factor model with two correlated superordinate factors to be acceptable models for the STARS [7,8]. The fit indices suggested Model 1 to be the best fit of the data compared to competing models. Nevertheless, the theoretical appropriateness of Model 1 is in question. Although Model 1 is useful in confirmatory factor analytic studies of the STARS, it does not make substantiative sense to have six correlated factors in research. For instance, the six factors of the STARS are often used with the explicit assumption that the factors are indicators of a higher level construct (i.e., statistics anxiety) [10,11]. Additionally, while part one discriminated between anxiety and attitudes, the Computation Self-Concept factor and the Fear of Statistics Teachers factor of part two had similar correlations with both anxiety and attitudes. Hence, we recommend researchers use Model 3 in their studies.

Model 3 appears to be a promising model. In terms of fit indices, it was a better model than Model 2, and had similar values on the CAIC and the ECVI with Model 1. With regard to theoretical appropriateness, Model 3 distinguished between anxiety and attitudes. This allows both variables to be studied concurrently and may provide researchers with clearer insights into their data. Therefore, we recommend researchers use part one of the STARS to assess statistics anxiety and part two to assess attitudes toward statistics.

Limitations of the study should be noted. First, the sample was drawn predominantly from psychology undergraduates in Singapore and Australia; the results might not generalize to graduate students or undergraduates in other disciplines (e.g., Information Technology). Second, the sample size did not permit separate investigations across demographic variables such as gender and campus/country (Singapore vs. Australia). In particular, since preliminary analysis found some differences in statistics anxiety for these variables, future research should examine the structure invariance of Model 3 across these variables.

The use of Model 3 provides several future research directions. Currently, the general consensus has been that negative attitudes toward statistics result in statistics anxiety [29]. Future research could administer both parts of the STARS at the start and end of the semester to test this notion empirically. Future research could also examine the relative importance of these two superordinate factors in predicting statistics achievement. Armed with such information, interventions could be designed to target the appropriate construct, either by reducing statistics anxiety or by reducing negative attitudes toward statistics.
